# Ensemble structure description of Lys63-linked diubiquitin^☆^

**DOI:** 10.1016/j.dib.2016.02.003

**Published:** 2016-02-09

**Authors:** Zhu Liu, Chun Tang

**Affiliations:** a Key Laboratory of Magnetic Resonance in Biological Systems, Wuhan Institute of Physics and Mathematics, Chinese Academy of Sciences, Wuhan, Hubei 430071, China; b Zhejiang University School of Medicine, Hangzhou, Zhejiang Province 310028, China

## Abstract

The data described herein are related to the article entitled “Lys63-linked ubiquitin chain adopts multiple conformational states for specific target recognition” [1], and to the coordinates for the ensemble structure of Lys63-linked diubiquitin (PDB code 2N2K). A Lys63-linked diubiquitin exists in three conformational states with different orientations for the two subunits, each responsible for binding to a target protein and encoding a specific cell signal. An atomic entry in the ensemble structure file consists multiple lines, representing alternative locations of the atom and recapitulating the dynamics of the protein. Experimental details about obtaining strictly intramolecular paramagnetic restraints and determining the relative occupancies of the conformational states are presented. The experimental design and procedures in this Data article can be useful for characterizing the structure and dynamics of other multi-domain proteins.

## 1 **Specifications table**

**Table:** 

Subject area	Biology
More specific subject area	Biophysics and structural biology
Type of data	Atomic coordinates, tab-limited text file
How data was acquired	NMR spectrometry (Bruker), ITC (GE Healthcare/Microcal), SAXS (Anton Paar, Graz, Austria)
Data format	Xplor-NIH input and PDB file
Experimental factors	Attachment of a paramagnetic tag at multiple positions of Lys63-linked diubiquitin for minimal structural perturbation
Experimental features	Integrate different biophysical techniques to characterize the ensemble structures of a multi-domain protein
Data source location	RCSB, Rutgers, NJ
Data accessibility	The ensemble structure for the multiple closed states of Lys63-linked diubiquitin along with NMR restraints has been deposited to the Protein Data Bank with accession code 2N2K

## 2 **Value of the data**

• Differentiate intramolecular versus intermolecular PRE contributions.
• Integrate NMR, SAXS and ITC for quantitative assessment of protein ensemble structure.
• Assess the relative occupancy for each conformational state of Lys63-linked diubiquitin.

## 3 Data, experimental design, materials and methods

### 3.1 Experiment design

Paramagnetic NMR, in particular paramagnetic relaxation enhancement (PRE), can provide long-range distance information and allows the depiction of protein conformational fluctuations [2]. Paramagnetic NMR is mostly performed with the conjugation of an extrinsic paramagnetic probe. As the linker between protein backbone and the paramagnetic center involves multiple rotatable bonds, the exact position of the paramagnetic center is not fixed with respect to the protein. The PRE value is related to the inverse sixth power of the distance between the paramagnetic center and a protein nucleus, and therefore a small fluctuation in the position for the paramagnetic center can cause a large variation in the PRE value. The observed PRE value for a protein nucleus in a certain conformational state is a product of the PRE values and the relative populations [3]. Thus it is difficult to deconvolute the occupancy of each protein conformational state from the back-calculated PRE.

Lys63-linked diubiquitin(referred to as Ub_2_) comprises two ubiquitins covalently linked via an isopeptide bond between Lys63 in one subunit and Gly76 in the other subunit, and is involved in many non-degradative signaling cascades in cell [4]. It has been found that Ub_2_ can exist in both open and closed conformations in solution [1], [5], [6]. Using the PRE, we characterized the structures of the two alternative closed states of Ub_2_. To assess the relative occupancies for each conformational state, we integrated the data from small angle X-ray scattering (SAXS) and isothermal calorimetry (ITC). SAXS data define the overall silhouette of the protein system, but lack structural details. ITC measures the binding affinities between Ub_2_ with its protein ligands. In a conformational selection mechanism, a point mutation (E64R in one subunit of Ub_2_) that perturbs the relative populations of the preexisting conformational states would affect the binding affinities towards the respective ligands. As such, the changes in binding affinities allow the extrapolation of the relative populations of the preexisting Ub_2_ conformational states.

### 3.2 Sample preparation

**Fig. 1:**
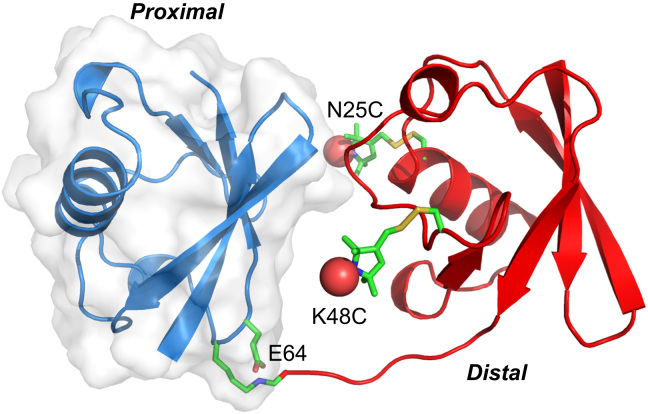
Illustration of the mutations introduced to Lys63-liked diubiquitin. The proximal subunit of Ub_2_ is shown as blue cartoon with transparent surface, and the distal subunit is shown as red cartoon. The MTSL paramagnetic probe conjugated at the engineered cysteine residue (one at a time) is shown as sticks, and the oxygen atoms carrying the unpaired electron are shown as red spheres. E64 in the proximal subunit where a charge reversal mutation (E64R) is introduced is also indicated.

UbiquitinTAB2 and Ub_2_ proteins were prepared according to the established protocol [7]. Specific ligands for Ub_2_, including tandem ubiquitin-interacting motif (tUIM), the fourth zinc-finger domain of A20 (A20 ZnF_4_), and the NZF domain of TAB2 protein (TAB2 NZF), were purified with GST affinity column followed by the removal of the GST tag with TEV protease. Single-point mutations were introduced using the QuikChange method. To specifically introduce the paramagnetic probe at a desired site, a Ub_2_ cysteine mutant (note that the mutations do not affect the enzyme-catalyzed formation of di-ubiquitin) was reacted with 5-times excess of S-(1-oxyl−2,2,5,5-tetramethyl-2,5-dihydro-1H-pyrrol-3-yl methyl methanesulfonothioate (MTSL, from Toronto Research Chemicals, Canada) for two hours at room temperature. Excess probe was removed through desalting, and the completeness of the conjugation was confirmed by mass spectrometry. The sites for paramagnetic conjugation (N25C and K48C both in the distal subunit of Ub_2_, one at a time) and single-point mutation (E64R at the proximal subunit of Ub_2_) are illustrated in Fig. 1.

### 3.3 Data

#### 3.3.1 Intramolecular PRE data

**Fig. 2:**
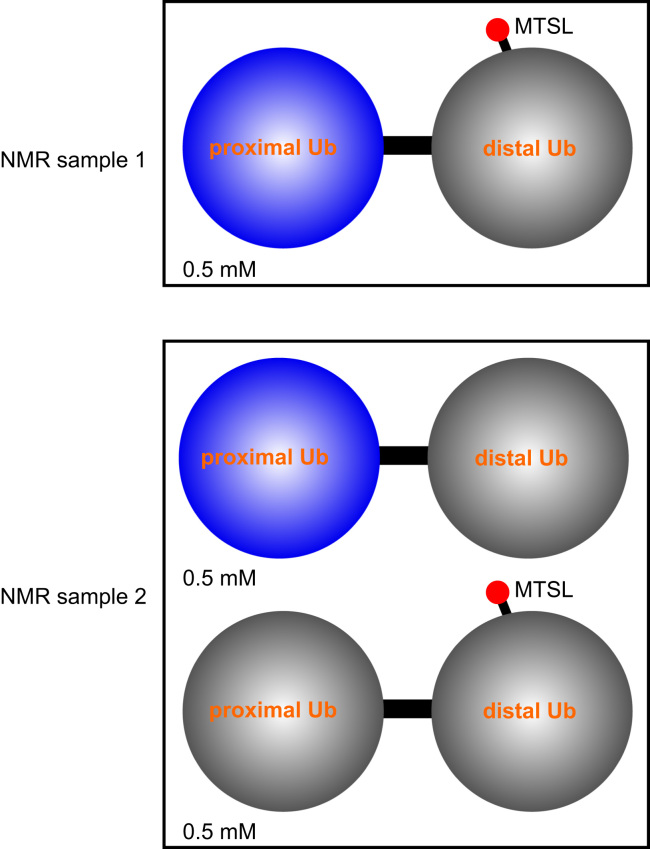
NMR samples used for PRE experiments. An MTSL paramagnetic probe is conjugated at either N25C or K48C site of the distal subunit of Lys63-liked diubiquitin. A blue sphere indicates the ^15^N-labeled subunit, while the gray sphere indicates the subunit with natural isotope abundance. PRE rates are measured for the ^15^N-labeled proximal subunit in both sample 1 and sample 2, and the differences are intramolecular PREs.

We used a two time-point NMR pulse scheme to measure the transverse relaxation rates for backbone amide protons [8], with a 4-ms delay between the two time points. In an Ub_2_ protein sample, one subunit is unlabeled and paramagnetically tagged, while the other subunit is [U-^15^N]-labeled for PRE measurement. The observed PRE rate for a particular nucleus can arise both intra-molecularly (between two subunits) or inter-molecularly (between two separate Ub_2_ molecules). Hence we prepared a second sample comprising an equimolar mixture of Ub_2_ proteins, with paramagnetic tagging and isotope labeling on different subunits of different Ub_2_ molecules (Fig. 2). The PRE values measured for the [U-^15^N]-labeled subunit in this mixed sample should only arise from inter-molecular, noncovalent interactions between different Ub_2_ proteins [6], which was subtracted from the PRE measured for first sample for strictly intramolecular inter-subunit PRE (Table 1).

#### 3.3.2 SAXS measurement

Small-angle X-ray scattering data were collected on a SAXSess mc^2^ instrument (Anton Paar, Graz, Austria) equipped with a sealed-tube source and a CMOS diode array detector. The proteins were extensively dialyzed. To remove any high molecular-weight aggregates, Ub_2_ was centrifuged at 15,000 rpm for 30 min prior, and the upper portion of the supernatant was carefully collected for SAXS measurement. To ensure the stability of the protein sample, SAXS data were collected every 30 min; the frames were compared, and were combined if there are no differences among them. As Ub_2_ adopts multiple conformations in solution, the SAXS data could not be fitted to any single known structure. The SAXS is rather insensitive to the transient noncovalent interactions between Ub_2_ proteins, and the data are the direct averaging (as opposed to <*r*^-6^> averaging for PRE) of the scattering profiles for the conformational states in solution.

#### 3.3.3 Binding affinities between Ub_2_ and its ligands

The binding affinity between wild type Ub_2_ (or the E64R mutant) and its cognate ligands (tUIM or TAB2 NZF) were measured on a VP-ITC instrument (GE Healthcare, Piscataway, NJ). The ligand protein (300 μM) was placed into a syringe and titrated into the reservoir containing wildtype or mutant Ub_2_ proteins (20 µM). The titration for each pair of interactions was repeated four times. The binding affinity between Ub_2_ and A20 ZnF_4_ was too weak to be assessed using ITC. Therefore, NMR titrations were performed on a Bruker 850 MHz instrument (Bruker, Billerica, MA), by titrating 50, 100, 150, 250, 350, 450 μM unlabeled A20 ZnF4 into 100 μM isotopically labeled Ub_2_ protein. The chemical shift perturbations on Ub_2_ could be globally fitted to a single-site binding isotherm.

A charge reversal mutation away from the binding interface to its partner protein was introduced at the interface between two ubiquitin subunits (Fig. 1). The E64R mutation destabilizes the preexisting closed-state conformations of Ub_2_ and promotes the open-state conformations. As a result, the binding affinity towards the open-state ligand (tUIM) increases, at the cost of decreasing binding affinities towards the closed-state ligands A20 ZnF_4_ and TAB2 NZF (Table 2; the binding affinities between Lys63-linked diubiquitin and A20 ZnF4 were obtained by fitting NMR chemical shift perturbations). Importantly, the changes in binding affinities are entropic, meaning that the mutation leaves the interaction between Ub_2_ and its ligands unperturbed and likely modulates the relative populations of the preexisting conformational states.

#### 3.3.4 Ensemble refinement of Ub_2_ structure

We refined the Ub_2_ ensemble structure against intramolecular inter-subunit PRE restraints using Xplor-NIH [9]. The paramagnetic probe was represented as three-conformer ensemble and was optimized. We fixed one subunit of Ub_2_ (the distal unit with paramagnetic probes conjugated), and allowed the other subunit (the proximal unit) to move as a rigid body, with torsional freedom given to the covalent linker between two subunits. The intramolecular PRE could not be fully accounted for with a single conformation for the Ub_2_ protein. Therefore we invoked ensemble representation, and we systematically incremented the number of conformers representing the closed state. The inter-subunit PREs could only be accounted for with a four-conformer representation for the closed state. However, the exact population of closed state was less certain, as the agreement between the observed and calculated PREs are all reasonably good at closed-state population >30%. Owing to the steep distance dependence of PRE, a small movement in the relative position of the two Ub units can compensate for the offset in population. At population <30%, however, the van der Waals repulsive term builds up, which would prevent further compensation and result in an disagreement between observed and back-calculated PREs.

The determination of the exact population for the closed state was based on several sources of evidences. (1) It has been shown previously that ubiquitin monomer noncovalently interacts with each other at an apparent K_D_ value of ~5 mM. When covalently linked with an isopeptide linkage, the effective concentration would drive ~70–80% of Ub_2_ to the closed state. (2) The SAXS profile is a direct average of the scattering profiles for the constituting conformers. In this case, it appears closer to the profile of the close state than to the profile of the open state. However, due to limited resolution, SAXS cannot tell whether multiple closed states exist or how the two Ub units interact with each other in the closed states. (3) A minimum of four-conformer ensemble is required to account for the intramolecular PREs. In the ensemble structure, three conformers are similar to each other, and are different from the other conformer. The four conformers could be clustered to two states (C1 and C2), and the number of conformers in each cluster represents the relative population and also serves as the scaling factor for the PRE. (4) The PRE profile for the E64R mutant is similar to that of the wildtype Ub_2_ protein, yet the magnitude is halved. This means the mutation decreased the closed-state population without perturbing the closed-state conformations. (5) The mutant proteins has decreased binding affinities towards the respective ligands for C1 and C2 closed states, and increased binding affinity towards the ligand for the open state. As the changes in binding affinities and binding free energies arise entropically (Table 2), the differences in conformational free energies, i.e. the changes in the relative populations of C1, C2 and open states, should be solely responsible for the changes in binding free energies. Using all the information above, we could determine the closed-state population at ~70%, and the open-state population at ~30%.

### 3.4 Description of the Ub_2_ ensemble structure

A concatenated file containing 70 refined structures was deposited at the PDB. The 70 structures in the PDB file are calculated with various starting coordinates, and are slightly different owing to structural convergence problem. With the distal Ub unit fixed, each structure comprises four conformers, representing alternative locations of the proximal Ub unit. As the Ub_2_ cannot be in all four locations at the same time, the atomic entries were given occupancy of 0.25 (the 9th column in the PDB file).

## Figures and Tables

**Table 1:** Intramolecular inter-subunit PRE data Lys63-linked diubiquitin.

	**N25C**			**K48C**		
*Sample 1*	*Sample 2*	*Intramolecular*^a^	*Sample 1*	*Sample 2*	*Intramolecular*^a^
2	3.9±0.6	3.7±0.6	0.2±1.2	7.5±0.4	5.6±0.4	1.9±0.8
3	4.4±0.7	5.0±0.8	−0.5±1.5	3.9±0.4	2.9±0.5	1.0±0.9
4	4.7±0.8	4.0±0.8	0.6±1.6	−0.2±0.5	−3.3±0.5	3.1±1.0
5	4.5±0.9	4.6±0.9	−0.1±1.8	8.6±0.5	4.0±0.5	4.6±0.9
6	10.3±0.9	7.6±0.9	2.7±1.8	13.1±0.5	5.4±0.4	7.7±1.0
7	12.3±0.7	7.2±0.7	5.0±1.4	15.0±0.5	5.8±0.4	9.2±1.0
8	75.8±4.9	56.8±2.8	19.0±7.7	90.9±7.2	85.8±5.8	5.1±12.9
9	21.6±1.1	10.6±1.0	11.1±2.1	55.8±2.0	35.2±1.3	20.6±3.3
10	22.5±0.7	5.0±0.6	17.5±1.3	34.8±0.7	11.5±0.4	23.3±1.1
11	24.3±0.6	5.4±0.4	19.0±1.0	46.9±0.9	10.7±0.3	36.2±1.2
12	75.5±2.9	8.8±0.7	66.7±3.6	54.8±1.5	6.8±0.4	47.9±2.0
13	6.3±0.8	3.5±0.8	2.8±1.6	19.1±0.7	8.6±0.7	10.5±1.4
14	15.1±0.7	3.5±0.7	11.6±1.4	20.1±0.5	3.0±0.4	17.1±0.9
15	4.4±0.7	4.3±0.7	0.1±1.4	4.7±0.4	2.4±0.4	2.3±0.8
16	4.1±0.7	3.4±0.7	0.7±1.4	19.4±0.5	12.4±0.4	7.0±0.9
17	2.5±0.8	3.4±0.8	−0.9±1.5	3.8±0.4	2.8±0.4	1.0±0.8
18	3.1±0.8	3.3±0.8	−0.2±1.6	1.0±0.4	−0.2±0.4	1.3±0.9
20	3.2±0.6	3.3±0.6	−0.1±1.2	6.0±0.4	3.9±0.4	2.1±0.8
21	3.1±0.5	3.1±0.6	0.0±1.1	3.6±0.4	1.6±0.4	1.9±0.7
22	1.5±0.7	1.8±0.7	−0.3±1.4	4.2±0.4	3.0±0.4	1.2±0.9
23	3.0±0.8	4.8±0.9	−1.9±1.7	4.2±0.5	3.1±0.5	1.0±1.1
25	2.8±0.6	3.2±0.6	−0.4±1.3	1.1±0.4	−0.2±0.4	1.4±0.9
26	2.8±0.6	2.3±0.6	0.5±1.3	4.3±0.3	3.7±0.3	0.6±0.6
27	3.0±0.7	4.3±0.8	−1.3±1.5	1.1±0.4	2.6±0.4	−1.5±0.8
28	2.5±0.5	2.3±0.5	0.1±1.0	4.7±0.3	1.3±0.3	3.4±0.6
29	2.6±0.6	3.7±0.6	−1.1±1.2	7.3±0.3	2.4±0.3	4.9±0.6
30	3.0±0.7	4.1±0.7	−1.2±1.4	3.3±0.4	3.0±0.4	0.3±0.8
31	2.0±0.7	2.8±0.7	−0.8±1.4	6.3±0.5	2.2±0.4	4.1±0.9
32	2.5±0.6	3.1±0.6	−0.6±1.1	11.8±0.4	2.0±0.3	9.8±0.8
33	3.3±0.5	3.2±0.5	0.1±1.1	12.4±0.3	4.1±0.3	8.3±0.6
34	3.9±0.9	3.8±0.9	0.1±1.8	7.0±0.5	3.6±0.5	3.4±1.0
35	5.0±0.9	7.4±0.9	−2.4±1.8	9.1±0.6	7.1±0.6	2.0±1.2
36	3.1±1.9	5.5±2.0	−2.3±3.8	3.3±0.5	3.6±0.5	−0.3±1.0
39	2.8±0.4	3.3±0.5	−0.5±0.9	3.6±0.3	4.3±0.3	−0.8±0.6
40	3.6±0.7	4.1±0.8	−0.5±1.5	2.1±0.4	3.8±0.4	−1.8±0.8
41	3.6±0.7	4.4±0.7	−0.8±1.4	4.3±0.4	3.6±0.4	0.7±0.8
42	3.9±0.8	4.7±0.9	−0.7±1.7	4.7±0.4	5.8±0.4	−1.1±0.9
43	5.6±1.0	4.3±1.0	1.3±2.0	6.2±0.6	4.9±0.6	1.2±1.3
44	17.1±0.9	10.3±0.9	6.8±1.9	16.3±0.6	8.8±0.5	7.5±1.0
45	17.7±0.9	9.7±1.0	7.9±1.9	21.1±0.8	7.5±0.6	13.6±1.4
46	38.1±1.5	14.7±1.1	23.4±2.6	2120±2000^b^	11.5±1.0	2108.5±2001.0
47	2120±2000^b^	50.9±1.5	2069.1±2001.5	2120±2000^b^	57.7±1.9	2062.3±2001.9
48	26.4±0.8	12.4±0.7	14.0±1.4	40.7±0.8	10.2±0.4	30.5±1.1
49	38.7±1.0	25.2±0.8	13.5±1.7	45.3±1.0	25.7±0.6	19.6±1.5
50	7.5±0.8	5.2±0.9	2.3±1.7	9.7±0.6	5.3±0.5	4.4±1.1
51	5.4±0.9	5.0±0.9	0.5±1.8	14.6±0.8	3.1±0.6	11.5±1.3
52	5.4±0.6	5.3±0.6	0.2±1.2	5.9±0.4	6.1±0.4	−0.2±0.8
54	2.8±0.6	3.7±0.6	−1.0±1.2	7.1±0.4	2.9±0.4	4.2±0.8
55	3.3±0.8	3.1±0.8	0.2±1.6	10.2±0.6	0.7±0.5	9.5±1.1
56	2.6±0.7	4.0±0.8	−1.3±1.5	5.5±0.5	3.4±0.5	2.1±1.0
57	3.4±0.5	3.9±0.5	−0.4±1.0	7.4±0.4	3.9±0.3	3.5±0.7
58	3.5±0.6	2.3±0.6	1.1±1.2	13.2±0.5	4.9±0.5	8.4±1.0
59	7.6±0.7	5.9±0.7	1.7±1.5	19.4±0.7	1.6±0.5	17.8±1.2
60	14.3±0.9	5.8±0.7	8.4±1.6	27.3±0.8	6.4±0.5	20.8±1.3
61	5.4±0.7	4.5±0.7	0.9±1.3	10.5±0.5	2.2±0.4	8.3±0.9
62	5.3±0.7	4.1±0.7	1.2±1.4	9.4±0.4	4.8±0.4	4.6±0.8
63	9.3±0.5	6.5±0.6	2.8±1.1	28.0±0.6	21.2±0.5	6.9±1.1
64	4.8±1.0	5.0±1.0	−0.2±2.0	4.1±0.5	2.8±0.5	1.3±1.0
65	3.9±0.5	3.7±0.5	0.2±1.1	5.9±0.4	3.1±0.3	2.8±0.7
66	16.6±0.8	10.4±0.8	6.2±1.6	40.3±1.6	15.3±0.8	24.9±2.4
67	6.4±1.1	4.4±1.1	2.0±2.2	8.2±v0.7	1.9±0.6	6.3±1.3
68	19.9±1.0	11.4±1.0	8.5±2.0	26.4±0.7	10.3±0.5	16.1±1.2
69	15.4±1.0	10.7±1.0	4.7±1.9	14.8±0.7	8.1±0.5	6.8±1.2
70	12.2±1.0	7.8±1.0	4.4±1.9	17.0±0.5	10.9±0.5	6.1±1.0
71	28.6±0.9	22.8±0.8	5.8±1.6	80.8±2.6	77.5±2.1	3.3±4.7
72	7.1±0.5	6.3±0.6	0.7±1.1	9.9±0.4	8.2±0.3	1.7±0.7
73	16.2±0.5	11.4±0.4	4.7±0.9	33.1±0.4	30.6±0.4	2.5±0.8
74	13.4±0.3	7.6±0.3	5.8±0.7	32.9±0.5	19.0±0.3	13.9±0.8
75	6.6±0.4	4.0±0.4	2.6±0.8	9.9±0.3	9.3±0.3	0.7±0.6
76	3.0±0.3	1.0±0.3	2.0±0.5	3.2±0.2	3.5±0.2	−0.2±0.3

a Error propagations are accounted for when subtracting the two sets of PRE data.

b The residue is broadened out beyond detection in the paramagnetic spectrum and gives a very large PRE value, with the lower limit at 120 s^−1^.

**Table 2:** Changes in *K*_D_ values (µM) between Ub_2_ and its partners upon mutation.^a^

	Wildtype				E64R mutant^b^			
*K*_D_ (μM)	Δ*H* (kCal/mol)	Δ*S* (Cal/mol/deg)	*N*	*K*_D_ (μM)	Δ*H* (kCal/mol)	Δ*S* (Cal/mol/deg)	*N*
NZF^c^	14.2±0.6	−15.0±0.5	−27.2	0.95±0.02	20.9±1.4	−15.0±0.9	−28.2	0.91±0.04
11.7±0.6	−12.0±0.3	−16.9	1.04±0.02	15.9±2.0	−12.4±1.1	−19.1	0.95±0.07
12.3±1.0	−12.4±0.7	−18.4	1.02±0.04	16.3±0.5	−11.7±0.3	−16.8	0.95±0.02
10.9±0.8	−11.3±0.5	−14.7	1.11±0.03	19.0±0.8	−13.1±0.5	−21.7	0.91±0.03
								
tUIM^c^	9.8±0.8	−21.3±0.9	−47.3	0.99±0.03	2.3±0.05	−21.3±0.2	−44.3	0.95±0.01
9.7±0.5	−21.8±0.7	−49.1	0.97±0.02	2.4±0.1	−21.3±0.3	−44.6	0.92±0.01
9.6±0.3	−23.2±0.3	−53.5	0.99±0.01	2.2±0.05	−20.7±0.1	−42.2	0.96±0.01
9.9±0.2	−22.8±0.3	−52.4	0.96±0.01	2.1±0.04	−20.5±0.1	−41.6	0.97±0.01

a The ITC measurements were repeated for four times at 303 K for either wildtype or mutant Ub_2_ proteins.

b By introducing the E64R mutation in the proximal unit of Ub_2_ protein, the binding affinities towards the respective ligands are affected, which are caused by changes in the Δ*S* values.

c The NZF is a ligand that specifically interacts with Lys63-linked diubiquitin in the C2 closed-state, and tUIM is a ligand that specifically interacts with Lys63-linked diubiquitin in the open state.
